# Education in mental health promotion and its impact on the participants' attitudes and perceived mental health

**DOI:** 10.1186/1744-859X-10-33

**Published:** 2011-12-23

**Authors:** Vlassis D Tomaras, Maria Ginieri-Coccossis, Maria Vassiliadou, Melpomeni Malliori, Spyros Ferentinos, Constantin R Soldatos, Andre Tylee

**Affiliations:** 1 Athens University Medical School, Athens, Greece; 2 Public Secondary Education Councelling Services, Athens, Greece; 3 Health Services Research Department, Institute of Psychiatry, King's College, London, UK

## Abstract

**Background:**

Although the promotion of mental health (MHP) through education and training is widely accepted, there is scarce evidence for its effectiveness in the literature from outcome studies worldwide. The present study aimed to assess the effect of a three-semester MHP educational program on the recipients' opinions towards mental illness and on their own self-assessed health.

**Methods:**

Respondents were 78 attendees who completed the assessment battery at the first (baseline) and the last session (end) of the training course. They were primary care physicians or other professionals, or key community agents, working in the greater Athens area. The course consisted of 44 sessions (4 h each), over a 3-semester period, focusing on the principles and methods of mental health promotion, the main aspects of major psychiatric disorders, and on relevant to health skills. Assessment instruments included the Opinion about Mental Illness (OMI) scale and the General Health Questionnaire (GHQ-28).

**Results:**

The mean scores of three OMI factors, that is, social discrimination, social restriction and social integration, and the two GHQ-28 subscales, that is, anxiety/insomnia and social dysfunction, were significantly improved by the end of the training course.

**Conclusions:**

The results of this study provide evidence, with limitations, for the short-term effectiveness of the implemented educational MHP program on an adult group of recipients-key agents in their community. Because interventions for strengthening positive opinions about mental illness and enhancing self-assessed health constitute priority aims of mental health promotion, it would be beneficial to further investigate the sustainability of the observed positive changes. In addition it would be useful to examine (a) the possible interplay between the two outcome measures, that is, the effect of opinions of recipients about mental health on their perceived health, and (b) the applicability of this intervention in individuals with different sociodemographic profiles.

## Introduction

For contemporary societies where the prevalence for mental disorders seems to compromise quality of life and economic prosperity, not only through direct costs of health and social services but also due to lost employment and productivity, the implementation of mental health promotion (MHP) programs is imperative as they may become instrumental for addressing such issues [1]. The Ottawa Charter, as a declaration statement and an institutional context, developed by the World Health Organization (WHO) in 1986, highlighted the goals of MHP and identified improvement of health policies as one of its first-line aims [2]. In the context of these premises, a MHP strategy can be advanced in every possible human setting by building healthy public policies, creating supportive environments, strengthening community action, developing relevant to mental health personal skills and in general reorienting health services to early detection of disorders and promotion of health and well-being [1,2]. Similarly, in the European Union, the so-called Green Paper was formulated in 2005, constituting a declaration document of proposals for the establishment of an inclusive strategy on MHP across the European countries [3].

It is widely recognized that promoting mental health and addressing mental ill health can be endeavored at different levels, taking into consideration individual, family, community and social determinants of mental health, and strengthening protective factors while reducing risk factors [1].

At a community and group level, the degree of knowledge and understanding about the nature of mental health and mental illness has been identified as a key element for changing health policies and practices [4]. Therefore, human settings such as schools and workplaces are considered crucial for MHP education. In schools in particular, an inclusive intervention program can increase social competencies, improve resilience and reduce bullying, anxiety and depressive symptoms [5,6]. In addition, promoting mental health in children, adolescents and parent populations may improve their mental health through the development of particular skills relevant to each group [7,8].

With regard to work environments, it is widely known that poor working conditions may lead to poor mental health, and increase sick leave rates and costs. Thus, interventions aiming at strengthening individual capacity and reducing stressors in the work setting are expected to improve health and support economic development [7].

It is also noted that the use of alcohol or any psychoactive substances constitute important MHP challenges to be addressed within different populations, not only in the high risk groups but also in early prevention programs, as for example in preschool children [3,8]. Targeting high-risk populations for ill health is a common methodological practice in MHP. Thus, in old age for instance, involving changes in the individual's functional capacity, social participation and mental health, supportive interventions aiming at improving and sustaining mental well-being in older patients are highly recommended [9]. In overall high-risk populations such as people with restricted socioeconomic resources, those experiencing job loss and unemployment, migrants and refugees or other marginalized groups constitute groups in need of supportive MHP interventions [3].

Besides MHP educational and training interventions for the enhancement of mental health in general, specifically addressing discriminatory attitudes and misconceptions about mental illness within the community is an important target of MHP actions. When negative and biased opinions are expressed within a particular social group, it is important to address such issues by means of MHP educational interventions at the level of community so that professionals and various groups of influence in the community may develop informed positive attitudes about mental illness [10]. In such cases, MHP programs may provide knowledge to key agents in the community as to recognize mental health issues, improving personal coping skills and becoming trained to initiate effective community action against ill health [11-13].

However, a major issue in policymaking regarding MHP programs concerns the effectiveness of interventions and evidence-based outcomes taking into consideration that examples of effective MHP activities are few and far between [14]. Tang *et al*. [15] proposed an interesting four-level typology of evidence in the field of health promotion. According to this typology, the first two types, types A and B, presuppose that what works and how it works in an intervention are known, while in the other two types, types C and D, what works is known but how it works is not known. Repeatability claims to be universal in type C, but limited in type D. Most interventions in the field of health promotion would be categorized within the lower levels of C and D evidence, while A and B levels are scarce. It is noteworthy that MHP actions take always place in specific and dynamic contexts, wherein sociocultural, political, human and coincidental or other factors may interact. In this sense, positive effects of a certain MHP intervention cannot be considered easily replicable at any time and any place.

In order to clarify the issue of how a MHP intervention works, distinction of the intervention's components and knowledge on causal links between them and the outcome measures are needed. In the absence of such knowledge, most of the MHP intervention programs can be classified as providing types C and D level of evidence.

Although scarce, examples of evidence based effective MHP educational programs in the literature do exist, a few of which are mentioned below. (1) An educational campaign carried out by Wolff *et al*. [16] on a target population neighboring supported houses for people with mental illness. The intervention led to a decrease of fearful and rejecting attitudes and increase of social contact with staff and patients. These findings suggest that the educational campaign exerted its effect on attitudes by encouraging contact with patients. (2) In New Zealand, Vaughan [17] reported on a nationwide MHP campaign, including education and training, providing evidence that awareness among the general public, as well as attitudes and behaviors towards people with experience of mental illness can be improved. (3) In a multisite European study (The European Early Promotion Project) [18], training was offered to a quasiexperimental group of primary health care professionals and its effects were assessed: it was found that recipients tended to improve their knowledge, perceived self-efficacy and ability to identify families in need. (4) Considering the dimension of cost benefits, Zechmeister *et al*., [19] extensively reviewed the existing research on the cost effectiveness of MHP and prevention interventions. They suggest that most favorable results belong, almost exclusively, to early intervention programs for children and adolescents, which thus seem worth financing.

In the present study we investigated the immediate or direct effects of a three-semester MHP intervention. The specific MHP educational program rests on providing scientific knowledge and information to key agents in the community regarding mental illness and mental health issues [11-13]. It includes addressing discriminatory attitudes and misconceptions about mental illness in the community and enhancing self-assessed mental health as they constitute important targets of MHP [10]. Therefore, the following hypotheses were tested in relation to this three-semester MHP program: (a) if the participants' opinions towards mental illness and the mentally ill individuals would become more favorable after receiving the MHP educational program, (b) if the participants' self-reported mental health would become more positive after intervention, and (c) if there would be any differences or changes in the variables of opinions and self-reported health according to sociodemographic variables.

## Methods

### The Athens MHP program

Starting in 2003, a specialized mental health educational program was implemented as part of a larger ongoing MHP project, developed by the Department of Psychiatry of Athens University in collaboration with the Section of Primary Care Mental Health, Institute of Psychiatry, University of London and the Educational Trust for Health Improvement through Cognitive Strategies (ETHICS), London [20]. The Hellenic Psychiatric Association was also a partner during the first year of implementation.

This MHP program was developed for providing to key community agents with fundamental MHP knowledge, addressing to their expressed needs to use it in their own professional settings and sociocultural networks. Specifically, the educational program was developed for providing scientific information and comprehensive knowledge about mental health and mental illness.

The syllabus of the educational course was informed by the relevant document of 'Axiological Promotion of Health' copyrighted by ETHICS [21] and was adapted to the sociocultural needs of the target group that is Greek community agents and professionals specialized and working in a variety of fields. A total of 44, 4-h educational sessions were organized and delivered over a 3-semester course, identified as the Athens MHP program, focusing on: (a) principles and strategies of mental health promotion and prevention of mental disorders; and (b) key points of the major and more common psychiatric conditions: anxiety, mood, somatoform and sleep disorders; substance and alcohol misuse and abuse; eating disorders; the interaction between physical and mental illness; child abuse and neglect; relational problems within and outside the family; conduct disorders in childhood and adolescence; psychotic disorders and stigma associated with them; dementia and its impact on carers.

The above topics were mostly addressed by academic lecturers with extensive clinical experience, who were asked to put emphasis on primary prevention, early identification and psychosocial aspects of disorders. Speakers were mostly from the Psychiatric Department of Athens University, and other institutions including the UK as well.

Besides the corpus of the above-mentioned lectures, an experiential MHP skills training unit including case studies was also provided focusing on development of coping strategies; management of stressful events, adversities and crises; developing resilience, personal autonomy and creative thinking; strengthening self-efficacy and identity of the self, as well as communication and family interaction skills.

In this part of the course participants were provided with pertinent MHP skills development activities, carried out within a workshop-style and task-oriented group setting. They were given the opportunity to bring relevant personal material to be discussed, to perform role playing and to present selected cases of mental health problems encountered within their own professional and social environments in order to receive supervision from assigned specialists/tutors. The cases included dysfunctional student behaviors in school, suicidal behaviors among privates during army service, burnout phenomena among health professionals. Thus, in the context of this workshop-style setting with an emphasis on promoting interactive group processes, the tutors facilitated a collaborative exploration of various MHP issues identifying efficient coping tactics for each case presented.

As indicated above, the effectiveness of this MHP program was tested by setting two hypotheses: a) regarding a change of opinions about mental illness becoming more favorable and b) enhancement of self-reported health. In a future application of this intervention, measures will be added to include detection of changes regarding dysfunctional conceptions about the self, others and the environment [22], as well as changes in subjective quality of life.

### Study design

The aim of the study was to test whether the implemented three-semester MHP educational program would bring about changes to the trainees towards more positive opinions about mental illness and mentally ill individuals and would also improve their self-assessed mental health status. To this end, trainees who were present and responded at the first and the last session of the training course, having full records of the two assessment points, were selected for statistical analysis (n = 78). The scores at baseline (BL) and end of the training (ET) assessments were compared and related to the respondents' sociodemographic characteristics.

### Subjects

For recruitment, invitations were addressed to various public agencies and selection of participants took place on the basis of expressed needs and personal motivation. Following a brief interview conducted by key members of the program, 121 candidates were selected. From those, 106 completed the educational program (23 physicians, 19 non-medical mental health professionals, 18 army and police officers, 13 educators, 12 judges and lawyers, 10 sociologists, 5 clergymen, 3 administrators, 2 journalists and 1 diplomat).

### Instruments

The administered instruments included the Opinion about Mental Illness (OMI) scale of Cohen and Struening, 1962 [23], and the General Health Questionnaire (GHQ-28) of Goldberg, 1978 [24].

The OMI scale investigates attitudes towards mental illness and consists of 51 items, in Likert format, with 6 alternative answers ranging from strongly agree to strongly disagree. Items can define five factors, that is: authoritarianism (A), benevolence (B), mental hygiene ideology (C), social restrictiveness (D) and interpersonal etiology (E). The Greek version of OMI scale, which was used in this study, also consists of 51 items assigned to 5 factors: *social discrimination *(A, 16 items: total scoring ranging from -14 to 66), *social restriction *(B, 13 items: total scoring ranging from -4 to 61), *social care *(C, 8 items: total scoring ranging from 30 to -10), *social integration *(D, 8 items: total scoring ranging from 33 to -7) and *etiology *(E, 6 items: total scoring ranging from 26 to -4). Factor analysis applied on a previous Greek survey's data identified these five dimensions, differentiated from the original OMI ones, with associated eigenvalues of > 1 and which accounted for 66.4% of the total variance in the data [25].

The GHQ is a self-report screening instrument of general health detecting non-psychotic psychopathology in clinical and non-clinical settings. The short form of GHQ consisting of 28 items was used in this study. Its four subscales measure *somatic symptoms*, *anxiety/insomnia, social dysfunction and severe depression*. The forms GHQ-60, GHQ-30 and GHQ-28 have been successfully tested for accuracy of translation and for their validity in the Greek context [26]. Since the GHQ scores did not serve any case identification purposes in our study, we decided to apply the Likert scoring method (that is, assigning 0, 1, 2, 3 values in each item) instead of the GHQ and C-GHQ scoring methods [27].

### Data analysis

First, it has been examined if there were significant sociodemographic differences between the group of 78 respondents and the group of 28 completers of the training course who presented missing values due to absence at either the first or second assessment, or both assessment times. Investigated variables included background characteristics: sex, age, years of education, marital status, type of education and previous MHP training experience. A χ^2 ^test for independence was performed on the aforementioned variables. Also, an independent samples t test was conducted in order to evaluate any differences in OMI and GHQ-28 scores between the two groups.

In order to assess the effect of the training program multivariate repeated measures analyses of covariance (MANCOVAs) were performed using questionnaire scores at the two time points as dependent variables. Cohen's effect sizes for the difference between the two time points were also calculated. Effect sizes of 0.2 to 0.3 were considered small and 0.31 to 0.5 medium [28]. Separate analyses were also conducted for the OMI and GHQ scores. Time (baseline and end of training), sex, age, length of education, marital status, type of education and previous MHP training experience were treated as covariate variables.

Correlation coefficients (Pearson r) were computed to evaluate the association between OMI and GHQ-28 scores. A *P *value of 0.05 was considered statistically significant. All statistical analyses were performed with the use of SPSS statistical software (V.16.0; SPSS, Chicago, IL, USA).

## Results

### Sociodemographics

The sociodemographic characteristics of 78 respondents were recorded. Women outnumbered men (48 vs 30); the total group's mean (SD) age was 38.8 (± 9.4) years; 42 were married, 29 were single and the remaining 7 were widowed, divorced or separated; their mean length of education was 17.4 (± 1.8) years; 43 were graduates of humanities and social sciences and 35 of sciences; a considerable number of them (32 out of 78) had some previous training experience in the field of MHP.

The 28 training course participants who did not complete both assessments did not qualify as respondents. Comparative analyses were run on these two groups (n = 28, n = 78). The two groups did not significantly differ on any of the aforementioned variables: sex (χ^2 ^= 0.9, *P *= 0.491); age (χ^2 ^= 1.7, *P *= 0.432); marital status (χ^2 ^= 0.8, *P *= 0.669); length of education (χ^2 ^= 1.7, *P *= 0.239); type of education (χ^2 ^= 0.4, *P *= 0.639); previous MHP training experience (χ^2 ^= 0.3, *P *= 0.647). They did not also differ on OMI and GHQ-28 scores at BL assessment: OMI factor A (t test, *P *= 0.801); OMI factor B (t test, *P *= 0.476); OMI factor C (t test, *P *= 0.960); OMI factor D (t test, *P *= 0.605); OMI factor E (t test, *P *= 0.776); GHQ-28 total score (t test, *P *= 0.768).

None of the aforementioned sociodemographic variables were found at BL assessment (n = 78) to have any association with the scores of the five attitudinal factors of the OMI scale. However, sex was found to affect the GHQ scores at BL assessment (n = 78): men (n = 30) had significantly lower total GHQ scores (that is, reported being healthier) than women (n = 48) (total GHQ-28 mean scores 14.4 and 18.6 respectively, *P *= 0.047), mainly due to significant scoring differences on the anxiety/insomnia subscale.

### OMI

The first repeated measures MANCOVA involved BL and end ET scores for OMI factors. As shown in Table 1, main effects of time indicated that *social discrimination*, *social restriction *and *social integration *significantly changed with respect to the time of assessment while the effect size of the differences were medium and ranged from 0.31 to 0.4. Respondents were demonizing mental illness significantly less and stigmatizing mentally ill patients at ET assessment less compared to BL assessment (*social discrimination *factor, mean scores 20.6 and 15.7 respectively, *P *= 0.040). They were also holding significantly less ostracizing and punishing stances towards the mentally ill and they believed less strongly that an individual with mental illness he (she) is incurable and in need of custodial care after their treatment (*social restriction *factor, mean scores 13.8 and 9.1 respectively, *P *= 0.041). They became significantly more tolerant and tended to recognize the psychiatric patient's equality in the role of employee (*social integration *factor mean scores 20.0 and 21.5 respectively, *P *= 0.036).

**Table 1:** Comparisons of Opinion about Mental Illness (OMI) scale and General Health Questionnaire (GHQ-28) mean scores in baseline (BL) and end of training (ET) assessments (n = 78)

	BL assessment, mean (SE)	ET assessment, mean (SE)	Effect size	F (df = 1.77)	*P *value^a^
OMI (Greek version):					
Factor A (social discrimination)	20.6 (1.9)	15.7 (1.3)	0.34	4.37	0.040
Factor B (social restriction)	13.8 (1.7)	9.1 (0.8)	0.40	4.31	0.041
Factor C (social care)	23.4 (0.5)	23.7 (0.4)	0.07	2.10	0.151
Factor D (social integration)	20.0 (0.7)	21.5 (0.4)	0.31	4.58	0.036
Factor E (etiology)	9.9 (0.7)	8.5 (0.6)	0.24	1.33	0.253
GHQ-28 subscales					
Somatic symptoms	4.1 (0.4)	3.4 (0.3)	0.22	3.29	0.074
Anxiety/insomnia	5.1 (0.5)	3.8 (0.4)	0.33	5.39	0.024
Social dysfunction	6.2 (0.3)	5.2 (0.3)	0.38	4.26	0.042
Severe depression	1.6 (0.3)	1.2 (0.3)	0.15	1.09	0.301
Total score	17.0 (1.1)	13.6 (0.9)	0.38	10.17	0.002

^a^*P *value for time effect (analysis of multivariate analysis of covariance (MANCOVA)).

### GHQ

Furthermore, the second repeated measures MANCOVA concerning GHQ-28 subscales showed some significant effects brought about by the training program. Specifically, scores on *anxiety/insomnia *(*P *= 0.024) and *social dysfunction *(*P *= 0.042) were significantly diminished at the end of the educational intervention (Table 1) with effect sizes equal to 0.33 and 0.38, respectively. As to GHQ-28 total score, it appeared to be significantly diminished at ET assessment (mean scores 17.0 and 13.6 respectively, *P *= 0.002), probably due to the significant differences noticed on the *anxiety/insomnia *and *social dysfunction *subscales' scores.

Table 2 presents mean changes in OMI factors with regards to all independent factors and the time × group interactions. Changes in the scores of the first three and the fifth OMI factors had a similar profile as far as the sociodemographic variables were concerned: sex, age, length of education, marital status, type of education and previous MHP training experience. Concerning factor D, a significant interaction of time with sex (*P *= 0.042) revealed that women benefited more from the intervention. Additionally, a significant interaction of time with length of education (*P *= 0.022) revealed that subjects with more than 18 years of education benefited more from the intervention in terms of factor D, compared to those with less than 18 years of education.

**Table 2:** Mean differences in Opinion about Mental Illness (OMI) scale scores between baseline (BL) and end of training (ET) assessments by levels of each independent variable (n = 78)

	Change criteria^a^		F (df = 1.76)	*P *value^b^
			
	Mean (SE)	Mean (SE)		
Sex	Men	Women		
OMI Factor A	-7.2 (3.1)	-3.4 (2.5)	1.50	0.225
OMI Factor B	-6.1 (2.7)	-3.9 (2.1)	1.43	0.236
OMI Factor C	-1.0 (0.8)	1.1 (0.7)	3.56	0.063
OMI Factor D	0.2 (0.1)	2.2 (0.8)	4.29	0.042
OMI Factor E	-2.0 (1.6)	-1.0 (0.9)	0.76	0.387
Age	≤ 39	≥ 40		
OMI Factor A	-7.4 (2.7)	-2.2 (2.8)	0.54	0.464
OMI Factor B	-7.4 (2.3)	-1.9 (2.3)	0.78	0.381
OMI Factor C	-0.3 (0.7)	0.9 (0.8)	0.66	0.420
OMI Factor D	1.3 (0.8)	1.6 (0.9)	0.14	0.706
OMI Factor E	-2.1 (1.5)	-0.6 (0.6)	0.19	0.733
Type of education	Theoretical	Exact sciences		
OMI Factor A	-5.8 (2.9)	-4.1 (2.6)	0.60	0.440
OMI Factor B	-4.6 (2.5)	-4.8 (2.2)	0.25	0.622
OMI Factor C	0.2 (0.8)	0.4 (0.7)	0.06	0.814
OMI Factor D	1.5 (0.9)	1.4 (0.8)	0.11	0.746
OMI Factor E	-1.1 (1.0)	-1.7 (1.3)	0.65	0.424
Previous MHP training experience	Yes	No		
OMI Factor A	-2.8 (3.0)	-6.3 (2.5)	0.10	0.751
OMI Factor B	-1.6 (2.5)	-6.9 (2.1)	0.01	0.929
OMI Factor C	0.5 (0.8)	0.1 (0.7)	0.01	0.950
OMI Factor D	2.1 (0.9)	1.0 (0.78)	0.83	0.366
OMI Factor E	-0.4 (0.6)	-2.0 (1.3)	0.17	0.681
Marital status	Married	Unmarried		
OMI Factor A	-3.0 (1.4)	-7.0 (3.9)	0.58	0.449
OMI Factor B	-2.2 (1.0)	-7.7 (3.3)	1.63	0.206
OMI Factor C	0.8 (0.6)	0.3 (0.9)	0.86	0.357
OMI Factor D	2.0 (0.6)	0.8 (1.1)	1.36	0.247
OMI Factor E	-0.7 (0.6)	-2.2 (1.6)	0.53	0.468
Years of education	≤ 17	≥ 18		
OMI Factor A	-7.7 (2.7)	-2.0 (2.7)	2.11	0.151
OMI Factor B	-7.9 (2.3)	-1.6 (2.3)	2.04	0.158
OMI Factor C	0.1 (0.8)	0.7 (0.8)	0.54	0.463
OMI Factor D	-0.5 (0.8)	2.4 (0.8)	5.46	0.022
OMI Factor E	2.5 (1.5)	-0.2 (0.7)	1.95	0.167

^a^change from BL to ET.

^b^*P *value for interaction effect with time (analysis of multivariate analysis of covariance (MANCOVA)).

Regarding changes in GHQ-28 scales with regards to the sociodemographic variables (Table 3) it was found that subjects aged above 40 years (*P *= 0.042 for interaction of time with age group) and those with more than 18 years of education (*P *= 0.049 for interaction of time with years of education) benefited less from the intervention concerning their *anxiety/insomnia *symptoms. Additionally, those with previous MHP training experience benefited more from the intervention in terms of their scores on the *social dysfunction *subscale.

**Table 3:** Mean differences in General Health Questionnaire (GHQ-28) scores between baseline (BL) and end of training (ET) assessments by levels of each independent variable (n = 78)

GHQ-28 subscales	Change^a^		F (df = 1,76)	*P *value^b^
			
	Mean (SE)	Mean (SE)		
Sex	Men	Women		
Somatic symptoms	-0.6 (0.4)	-0.8 (0.5)	0.02	0.895
Anxiety/insomnia	-1.0 (0.6)	-1.4 (0.6)	0.08	0.778
Social dysfunction	-0.8 (0.6)	-1.2 (0.6)	0.49	0.487
Severe depression	-0.6 (0.4)	-0.4 (0.3)	0.24	0.623
Age	≤ 39	≥ 40		
Somatic symptoms	-0.7 (0.6)	-0.8 (0.4)	0.10	0.750
Anxiety/insomnia	-1.7 (0.7)	-0.8 (0.6)	4.33	0.042
Social dysfunction	-1.1 (0.5)	-0.9 (0.6)	0.19	0.664
Severe depression	-0.8 (0.3)	-0.1 (0.4)	0.16	0.687
Type of education	Theoretical	Exact sciences		
Somatic symptoms	-0.8 (0.5)	-0.7 (0.5)	0.03	0.859
Anxiety/insomnia	-1.0 (0.6)	-1.5 (0.7)	1.61	0.208
Social dysfunction	-0.9 (0.6)	-1.2 (0.5)	0.09	0.771
Severe depression	-0.4 (0.4)	-0.5 (0.3)	0.04	0.844
Previous MHP training experience	Yes	No		
Somatic symptoms	-0.9 (0.4)	-0.6 (0.5)	0.39	0.533
Anxiety/insomnia	-1.3 (0.4)	-1.2 (0.7)	0.43	0.516
Social dysfunction	-1.8 (0.5)	-0.5 (0.6)	4.41	0.038
Severe depression	-0.4 (0.3)	-0.4 (0.3)	1.09	0.301
Marital status	Married	Unmarried		
Somatic symptoms	-0.7 (0.3)	-0.8 (0.6)	0.01	0.964
Anxiety/insomnia	-1.1 (0.5)	-1.4 (0.8)	0.02	0.878
Social dysfunction	-1.1 (0.4)	-0.9 (0.7)	0.04	0.849
Severe depression	-0.5 (0.3)	-0.4 (0.4)	0.45	0.507
Years of education	≤ 17	≥ 18		
Somatic symptoms	-1.0 (0.6)	-0.5 (0.4)	0.76	0.386
Anxiety/insomnia	-1.9 (0.7)	-0.6 (0.5)	3.97	0.049
Social dysfunction	-0.5 (0.7)	-1.5 (0.4)	0.71	0.401
Severe depression	-0.4 (0.4)	-0.4 (0.3)	0.08	0.785

^a^change from BL to ET.

^b^*P *value for interaction effect with time (analysis of multivariate analysis of covariance (MANCOVA)).

MHP = mental health promotion.

### Relationships between OMI and GHQ mean scores from baseline to the end of training

The correlations between the difference of OMI factors (A, B, C, D, E) from baseline to end of training and the respective difference of GHQ-28 total score were not statistically significant: dif OMI-A factor by dif GHQ-28 Total score, Pearson r 0.151, (NS); dif OMI-B factor by dif GHQ-28 Total score, Pearson r .133,(NS); dif OMI-C factor by dif GHQ-28 Total score, Pearson r.035,(NS); dif OMI-D factor by dif GHQ-28 Total score, Pearson r.206,(NS); dif OMI-E factor by dif GHQ-28 Total score, Pearson r .08, (.NS).

## Discussion

As indicated by Tang *et al*. [15], proposing a four-level typology of evidence, the present study may provide some evidence regarding the effectiveness of the implemented MHP intervention, and this evidence can be classified into the types of C and D according to this model. Taking into consideration the findings showing medium effect size in OMI and GHQ-28, the results may suggest that there is some improvement at a significant level, as reported by the participants regarding their opinions about mental illness and their self-assessed health. The results fail to provide evidence classified into Tang *et al*.'s A and B types as there is no certainty with regards to how exactly this positive change was brought about and which element(s) of the intervention have induced improvement. It may be assumed that the knowledge acquired in the specific MHP program may facilitate changes following evidence of the relationship in other studies [29].

Concerning the profile of the participants, no differences were found between those who responded in the questionnaires (N = 78) and those who did not complete both assessments (N = 28), suggesting that the reported positive outcomes were related to the intervention rather than to individual characteristics of the participants. Sociodemographic variables, that is, gender, age, education and sciences vs humanities and social sciences, marital status and previous MHP training, did not seem to be associated with the opinions held at baseline about mental illness. In terms of perceived health, women, however, reported experiencing more anxiety symptoms.

In general, the participants of the specific MHP three-semester course significantly improved their opinions towards mental illness and the mentally ill, a fact which was reflected in improved values in the OMI factors A, B and D, that is *social discrimination, social restriction *and *social integration*. Notably, there was no change on factor C referring to *social care *(Table 1). Considering that factor C consists of items eliciting views about mental health and social care policy, a possible interpretation might be that such views are rather stable among participants and cannot be influenced by the MHP intervention.

The findings of the present study on opinion changes are in agreement with other studies showing the effectiveness of educational campaigns and training in decreasing rejecting attitudes in the participating populations while increasing social contact with mentally ill patients [16,17]. These findings support our general hypothesis that MHP educational interventions may exert a beneficial effect on improving opinions and hopefully behaviors towards people with mental illness and encouraging contact with them.

In general, improvement on OMI scores after a relevant MHP educational intervention would be expected. It is remarkable that the sample's BL scores on the first four OMI factors were significantly better compared to those reported in two previous community surveys in an Athens district. The first, conducted in 1980, had reported mean values such us 41.8 (± 9.4) for factor A, 27.1 (± 10.1.) for factor B, 24.6 (± 3.4) for factor C and 16.2 (± 5.0) for factor D [25]. The second, conducted in 1994, reported 35.1 (± 6.6), 23.8 (± 6.8), 22.4 (± 2.3) and 12.5 (± 5.4) respectively [30]. The discrepancy between these earlier findings and the results of the present study can be attributed to different factors such as the attitudinal differences which are to be expected between groups of the general population and the sample of the present study consisting of individuals with higher educational and professional status. Another cause could be the community mental health awareness enhanced through education, which has taken place in Athens greater area in the meantime.

Regarding the noted improvement in perceived mental health, the GHQ-28 scores of the sample at baseline assessment were found within the normal range of healthy individuals, since the cutoff 23/24 which is indicated for the Likert method [27] is far above the sample's mean values (17.0 ± 9.8). Comparisons cannot be made as the relevant studies referred to clinical studies using an alternative method of scoring and proposing thus cut off points of 5/6 [31]. It is argued that baseline scores of GHQ-28 subscales of *anxiety/insomnia *and *social dysfunction *becoming significantly lowered by the end of the intervention may be interpreted as an indication that this intervention might have improved subjective well-being and perceived health status (Table 1), although the existence of a control group would strengthen the aforementioned results. Also, it is of note that it is not possible to know the exact mechanisms of these changes, so at least two questions can be raised: to what extent the observed improvement in perceived mental health is mediated by cognitive inputs due to learning through education? Might this effect be attributed to the specific educational setting and the supportive and cohesive group that has operated as such for three consecutive semesters? An interaction of these variables may also strengthen the outcomes.

Concerning sociodemographic variables on improvement of opinions with the end of the intervention (Table 2), it was found that in factor D of the OMI, women and participants with more than 18 years of education appeared to have benefited more from the intervention recognizing the need of the mentally ill for equality in the role of employee (*social integration *factor).

As for changes in perceived mental health in the GHQ-28 scales (Table 3), it appeared that subjects aged above 40 years and those with more than 18 years of education benefited less from the intervention concerning their *anxiety/insomnia *symptoms. Additionally, those with previous MHP training experience benefited more from the intervention in terms of their scores on the *social dysfunction *subscale.

Taking into consideration that these findings seem to support the hypothesis that beneficial changes could be initiated by this MHP educational program, it could be argued that the implemented intervention may be fruitful to other groups of professionals and community agents.

Besides applicability, there are aspects of the MHP intervention outcome which require further attention: (a) durability of changes found at ET assessment, and (b) the utilization of these positive changes (regarding opinions about mentally ill individuals and perceived mental health) beyond the direct effect of the intervention on the participants. It could also be assumed that improvement of self-reported health may also imply benefits for quality of life, so a future investigation might include an investigation of this relationship and assessment of the effect of the program on participants' quality of life with the use of generic and health related quality of life validated tools [32].

A final point concerns the importance placed on investigating and evaluating how the participants of the program utilize the acquired skills and convey the MHP benefits into their familial, occupational and social environments, especially into their work settings. To this end, the coordinators of the MHP project have planned a qualitative study of the recipients in order to monitor the expected implementation of their newly acquired MHP skills, within all the aforementioned contexts, as well as their sustainability. Keeping in mind that this is very difficult to measure, other types of 'internal' evidence such as personal examples and case reports are going to be used.

## Limitations

The main limitation of this study is the lack of control group(s), restricting the power of evidence regarding the program's effectiveness and leaving some important questions unanswered. As indicated earlier, it would be interesting to know whether it was the lecturing on well selected topics in mental health given in the first part of the three-semester MHP program or the workshop group activities in the final part including the presentation and discussion of real cases that produced the above-mentioned changes. The design of this study did not allow for comparisons between these components of the intervention so as to identify the most influential factors bringing about possible improvement.

The changes in self-reported health constitute another limitation as these could be due to other factors besides the educational intervention. One is selection into the course of people with slightly higher GHQ scores and subsequent regression to the mean. Another possibility is a retest artifact. Symptom scales tend to give lower scores on retest, even where there has been no plausible change. A third is an increase in social desirability for certain responses following the course.

## Conclusions

Developing positive attitudes regarding mental illness and mentally ill individuals seems to be related to the specific MHP educational intervention. Furthermore, improvement in self-reported health seems to be possible due to such intervention. While taking into consideration that including a control group would strengthen the results, it is however important to notice the effectiveness of such programs in accomplishing basic MHP aims in the community, such as the promotion of antistigmatizing attitudes for mental disorders and enhancement of self-reported health.

## Competing interests

The authors declare that they have no competing interests.

## Authors' contributions

VT: design, interpretation, preparation of manuscript, editing, revising. MGC: design, interpretation, preparation of manuscript, revising, editing. MV: design, data collection, interpretation. MM: design, comments on first draft. SF: statistical analysis. CS: interpretation, editing. AT: overall coordination.
